# Target templates specify visual, not semantic, features to guide search: A marked asymmetry between seeking and ignoring

**DOI:** 10.3758/s13414-016-1094-7

**Published:** 2016-04-07

**Authors:** Jennifer L. Daffron, Greg Davis

**Affiliations:** Department of Psychology, University of Cambridge, Downing Street, Cambridge, CB2 3EB UK

**Keywords:** Visual search, Object recognition, Object-based attention

## Abstract

Top-down search templates specify targets’ properties, either to guide attention toward the target or, independently, to accelerate the recognition of individual search items. Some previous studies have concluded that target templates can specify *semantic* categories to guide attention, though dissociating the effects of semantic versus *visual* features has proven difficult. In the present experiments, we examined the roles of target templates in search performance, by measuring the “two-template costs” incurred when observers did not know which of two types of targets would be presented. For target templates, these costs only varied with set size when a template could specify a target’s features. Any semantic influences did not affect the guidance of attention, only the recognition of individual items. In contrast, templates for rejection—specifying the properties of irrelevant nontargets—*do* appear to specify semantic properties to guide attention away from those items, without affecting recognition. These qualitative differences between the two types of templates suggest that the processes of seeking and ignoring are fundamentally different.

To understand human visual search, we must unravel its interwoven influences of bottom-up segmentation, evidence accrual, and competition, from top-down guidance and criterion setting. Some of Professor Yantis’s most valued and enduring contributions to our understanding of attention have tackled this issue head-on, isolating those effects forced upon the observer by the stimulus from voluntary, goal-directed control. In earlier studies, Yantis made a strong case for the ability of some bottom-up, stimulus-driven features, particularly sudden onsets, to override top-down settings (Remington, Johnston, & Yantis, 1992; Yantis, 1993; Yantis & Jonides, 1984, 1990). Conversely, in other work (e.g., Greenberg, Esterman, Wilson, Serences, & Yantis, 2010), he isolated the top-down influences of target templates, measuring *prestimulus influences* in high-level, category-specific visual cortex. Our work here, and in this literature as a whole, owes a debt of gratitude to Yantis.

Our primary goal in designing these four new experiments was to help resolve whether top-down search templates can guide attention toward target objects on the basis of their semantic features, or only of their visual features, in real-world search tasks. When, for example, an observer wishes to detect a person in a natural scene, can their search template only bias attention toward the target by specifying human beings’ visual features, or can their template specify those objects’ semantic properties to guide attention? This ambiguity does not arise for many conventional visual search studies, which examine search for simple abstract images, but it is particularly prominent in heterogeneous and complex naturalistic object images. Although abstract search items provide optimal control of variation in the stimulus properties, “real-world” search may operate differently, given the much greater complexity and diversity of object images in natural scenes (e.g., Võ & Henderson, 2010). Accordingly, to better approximate some key features of real-world search, many recent visual search experiments have employed photorealistic images of objects, scene backgrounds, or even video. Under these (perhaps more challenging) conditions, some previous studies have concluded that establishing a search template to look for one category of objects also biases attention to semantically related irrelevant objects. Such conclusions challenge influential models of search (e.g., Wolfe, Alvarez, Rosenholtz, Kuzmova, & Sherman, 2011) that have assumed that search templates guide attention by specifying *bundles of visual features* associated with targets. Moreover, findings of successful semantic guidance are difficult to integrate with other findings from the visual search literature that have shown that search for objects based on their semantic categories is typically inefficient (Purcell, Stewart, & Skov, 1996; Wolfe & Bennett, 1997).

## Can target templates guide search on the basis of semantic features?

Many “real-world” search tasks involve search for a category of objects—keys, fruit, shoes—rather than for a specific target object image. In such cases, the ability to specify that category of objects with a top-down search template to guide attention to the sought category would be invaluable. A growing corpus of work has attempted to reflect these aspects of real-world foraging in laboratory search tasks. These studies present photorealistic images of objects, sometimes in natural scene backgrounds, and compare the efficiencies of search for objects on the basis of being asked to find (1) a specific image or (2) a target defined only by a verbal label. The results from this work have established, first, that search based on a label (e.g., “trousers”) is possible, and, second, that search for a specific image is generally more efficient than search based on a label (Maxfield & Zelinsky, 2012; Schmidt & Zelinsky, 2009; R. Wu et al., 2013). However, whether attention may be guided toward sought items on the basis of their semantic features/category membership, rather than of sets of associated visual features, remains uncertain.

As we discuss here, in generating evidence for semantic/category-based guidance, it has proven very difficult to preclude explanations based on guidance due to visual features (e.g., Yang & Zelinsky, 2009). Note that we do not consider the guidance of attention by scene gist—not because we think it need be an exception to the conclusions drawn here, but rather because in natural scene backgrounds, spatial associations are very difficult to disentangle from guidance by the semantic properties of objects (Hwang, Wang, & Pomplun, 2011; C.-C. Wu, Wick, & Pomplun, 2014). Furthermore, as Wolfe et al. (2011) have noted, it is difficult, perhaps impossible, to specify the “set size” (number of search items) in natural scenes. Without a definitive “set size,” there is little basis to distinguish semantic influences on guidance of attention from semantic influences on object recognition. In terms of reaction time (RT) analyses, only when set size is manipulated is there the potential to distinguish influences on the guidance of attention from influences on the recognition of individual items in a search display.

From studies in which set size has been formally manipulated, the previous evidence has often been consistent with guidance by target templates specifying either semantic *or* visual features. For instance, Yang and Zelinsky (2009) asked participants to search for a category of object images, noting that this was relatively efficient, but also noted that a machine classifier could distinguish that target category from nontarget items on the basis of its features. Accordingly, those results could not distinguish guidance by visual features from guidance by semantic properties of the target objects. Similarly, Reeder and Peelen (2013; see also Reeder, van Zoest, & Peelen, 2015) have demonstrated that when observers establish a template to search, for example, for people in a display, their attention will also tend to be guided toward silhouettes of people, and not toward other silhouettes. Although it is possible that such attention might have been directed toward silhouettes due to their membership in a particular category, the details of those results have tended to weigh against such an explanation. Reeder and Peelen found that performance generally, and the biasing of attention to silhouettes specifically, was not affected by inverting the silhouettes; in contrast, Stein, Sterzer, and Peelen (2012) showed that the efficiency with which silhouettes are attributed to a category *is* affected by inversion. Accordingly, Reeder and Peelen’s results also seem more consistent with guidance of attention toward a set of features associated with an object category, rather than toward the semantic properties of that category.

Some of the most compelling evidence for semantic guidance of attention has addressed the spontaneous misallocation of attention toward nontarget items that are semantically related to, though visually dissimilar from, the specified target item (e.g., Belke, Humphreys, Watson, Meyer, & Telling, 2008; Moores, Laiti, & Chelazzi, 2003). Those semantically related nontargets were also more likely than semantically unrelated nontargets to be fixated first during search, particularly when no target was present; this effect was independent of the number of objects in the search display (in Belke et al., 2008). Subsequently, however, Malcolm and Henderson (2009) noted (specifically in relation to Vickery, King, & Jiang, 2005, and Wolfe, Horowitz, Kenner, Hyle, & Vasan, 2004) an issue in those studies that might threaten these conclusions. In these studies, the target and nontarget stimuli were each presented on multiple occasions during the experiment. Additionally, observers were instructed to study all of the objects used in the experiment before the experiments began, raising the possibility that attention had been guided to the specific, remembered, semantically related images in those studies, rather than to broad categories of image. Accordingly, in our experiments, we sought only to present each target (or nontarget) once during an experiment, to minimize this issue. A further factor that may complicate interpretation of these findings (Moores et al., 2003) is that the initial fixations were predominantly on targets and had the same latency as recorded first saccades to related nontargets; this suggests that by the time observers were making initial saccades toward related nontargets, they had largely completed all of the processing to know whether the target was present and its location.

### A barebones hybrid search task

Our brief survey of the literature, outlined above, convinced us that, although the balance of evidence was consistent with the notion that target templates could guide search by specifying semantic features, substantial further support would need to be garnered to establish this securely. To address this issue, here we employed a “barebones” version of Wolfe and colleagues’ hybrid search task (e.g., Wolfe, 2012), in which the key displays comprised just two items—one target object and one nontarget. This simple paradigm eliminated the complicating factor of nontarget–nontarget interactions and “odd-one-out” effects that likely drive the bottom-up, stimulus-driven guidance of attention (there can be no physical or semantic “odd one out” of two items). Within these trials, two effects of target templates could influence performance: (1) effects of the guidance of attention and (2) effects on recognition of the target as a target (e.g., Maxfield & Zelinsky, 2012). To help distinguish these two influences, we made one further necessary addition to the task, by including some trials on which only the single target was presented. In that condition, we assumed that guidance-of-attention effects due to a search template should be minimal, since attention would be drawn immediately to the single search item. Consequently, any effects of search templates that arose *only* for the two-item displays would likely reflect the guidance of attention. Effects that arose for both the one- and two-item displays would likely include influences of the template on the *recognition* of the target as a target item, rather than on the guidance of search. To make the task a simple “present” versus “absent” decision about the target, we also included one- and two-item trials on which only (one or two) nontargets were presented; the role of guidance in determining performance in either of these conditions, of course, would be uncertain, since there was no target to which to be guided.

With this basic logic in place, we could then manipulate the top-down templates that observers had to establish and apply. Our primary manipulation in the experiments reported here was to specify in some blocks of trials that the target could be *either of two* possible types of target object (in Exp. 1A, either a clock or a key, randomly assigned depending on the trial), but to specify in other blocks of trials that the target would be of one possible type (in Exp. 1A, segregated blocks of only key or only clock targets). The basic notion was that if, on the former type—“two-template” trials—observers could establish and apply two separate templates independently in parallel (i.e., a template for “key targets” paired with a template for “clock targets”; see, e.g., Barrett & Zobay, 2014), they could perform equally well as in the latter, “one-template” type of trial (i.e., only a “key target” template was necessary; see Wilschut, Theeuwes, & Olivers, 2013, for a similar logic applied to simple, abstract stimuli). We refer to a slowing of RTs on two- versus one-template trials as a “two-template cost.” This cost is referred to throughout our experiments and was calculated by subtracting the mean RT of one-template trials from the mean of two-template trials. Although this term might feel a little theory-laden, other obvious choices have either been applied to different circumstances (a “two-target cost” refers to conditions in which two targets appear in the display) or are not sufficiently general (“two-category cost” does not capture within-category target variation, as in Exp. 4).

The task described here measures two-template costs, holding the stimulus sets constant across two-template and one-template blocks of trials (though the specific images were not repeated); only the instructions changed. For instance, RTs to detect the presence of a clock image target, accompanied by a nontarget image, can be measured under conditions in which the observer knows that the target will be a clock (and needs to establish only that search template) versus when the observer knows that the target may be *either* a clock *or* a key (and would ideally establish those two templates independently in parallel). Any costs in the latter case, relative to the former, we assume would reflect a general difficulty with holding or applying two templates efficiently in parallel.

In a previous study of “templates for rejection” (i.e., search templates that specify nontargets’ properties to suppress attention to those items), we found that two-template costs were very robust (Daffron & Davis, 2015). That finding was consistent with Wolfe’s “log law” of hybrid search—that search becomes broadly less efficient with increasing numbers of potential types of targets (e.g., Wolfe, 2012). However, when the two nontargets were drawn from physically dissimilar, but *semantically related*, categories (e.g., locks and keys), no two-template costs were observed. We attributed the absence of such costs (a potential violation of Wolfe’s log law) to the ability of templates for rejection to specify the semantic features of nontargets. Some semantic features were presumably shared when the two possible categories of nontargets were semantically related, and thus, by specifying those overlapping properties, a single template for rejection could serve to specify both categories of nontargets and obviate any need to hold two templates concurrently. Hence, there would be no two-template cost. A striking feature of two-template costs in relation to templates for rejection was their absence in one-item displays, even for unrelated categories of targets—reflecting changes in the guidance of attention, rather than in the recognition of (non)targets as such.

In this set of experiments, we applied that same logic to the study of target search templates, which specify the properties of targets in order to enhance guidance of attention toward those objects and speed recognition of them as targets. Prior to running any such experiments, we were confident that the same patterns would emerge as for templates for rejection. However, an initial foray into this topic (Exp. 6 of Daffron & Davis, 2015) had produced no evidence of semantic effects on target templates, leading us to question those assumptions. To anticipate the findings described here, the experiments described in this article strongly support the core assumption of Wolfe’s (2007; Wolfe et al., 2011) Guided Search model, that (potential influences of scene schemata aside) target templates bias attention toward targets on the basis of (“loose bundles” of) associated visual features, not semantic features.

In Experiments 1A and 1B, we examined two-template costs when observers were asked to search for target photographs comprising pictures of either clocks, keys, or locks. In Experiment 1A, the target item was always a clock or a key (i.e., drawn from either of two semantically unrelated categories), and in Experiment 1B, a lock or a key (i.e., two physically different, yet semantically related categories). The crucial manipulation, as described above, was that on some blocks of (one-template) trials, the observer knew what the target’s category would be, but in the remaining (two-template) blocks, the observer knew only that the target could be from either of the two categories.

If search templates can specify the semantic properties of targets to guide search (and particularly if, as previous work has concluded, the template tends to spread to enhance attention toward semantically related categories), we should expect to find that the observers in Experiment 1B could reduce/abolish two-template costs by selecting with their search template the semantic features shared by the two categories. This should yield smaller two-template costs in Experiment 1B than in Experiment 1A, and perhaps no evidence of a two-template cost. Though such a finding might seem counterintuitive, we found it in three out of three experiments for templates for rejection (Daffron & Davis, 2015). The absence of any difference between the two-template costs for Experiments 1A and 1B would therefore also be a salient signal of minimal semantic contributions to the effect.

## Experiments 1A and 1B

Experiments 1A and 1B measured RTs for detecting a single, predefined target category versus for two potential target categories, to reveal two-template costs associated with semantically related or unrelated target categories. Instructions preceding the initiation of each block determined the necessary number of target templates to be implemented by revealing the target(s) for the following set of trials. The exact features of the target remained unspecified; observers were only instructed on the target image’s category. Nontargets in these studies could be any image other than the potential target categories (a shoe, book, chair, etc.).

### Method

#### Observers

Forty observers (Exp. A, ten male, ten female, aged 18–28 years; Exp. B, ten male, ten female, 18–29 years of age) from the University of Cambridge and the local area gave written informed consent and were paid for participating.

The effects of interest in this first study had generated effect sizes from templates for rejection (Daffron & Davis, 2015) of Cohen’s *f* = 0.366 (Exp. 3; here we confirmed this estimated effect size with a different task, finding Cohen’s *f* = 0.359). With a liberal alpha of .1, a sample size of 40 observers should, given standard assumptions, yield power of around 90% to see an effect corresponding to that observed for templates for rejection (based on G*Power 3: Faul, Erdfelder, Lang, & Buchner, 2007).

#### Materials

The experiment was conducted on a Mac Mini processor presenting on a Dell P2414HB LCD Monitor with a panel size of 60.97 cm at its optimal resolution of 1,920 × 1,080 at 60 Hz. The experiment was programmed and run in PsyScope XB57 (Cohen, MacWhinney, Flatt, & Provost, 1993). An attached Apple keyboard was used for observer responses. The observers in Experiment 1A (semantically unrelated target categories) viewed displays comprising 144 randomly selected neutral nontarget images, 72 target images of keys, and 72 target images of clocks. The observers in Experiment 1B (semantically related target categories) viewed 144 randomly selected neutral nontarget images, 72 target images of keys, and 72 target images of locks. Observers were positioned at a comfortable viewing distance of about 50 cm, and images were presented in 150 × 150 pixel-sized boxes (35 × 35 mm) centered 32.5 mm above, left, below, and right of fixation.

#### Procedure

The experiment began with an instruction screen detailing the broad structure of the experiment. Observers learned they would see three target categories of images—keys and clocks (Exp. 1A)/locks (Exp. 1B), and nontarget “neutral” images (drawn from a broad range of objects that did not include the target categories or related objects)—and the nature of their task, which was to detect whether or not a target item was present on each trial. An additional instruction screen appeared before the initiation of each block, to indicate the specific target/targets the observers were tasked with finding for the following block.

**Fig. 1:**
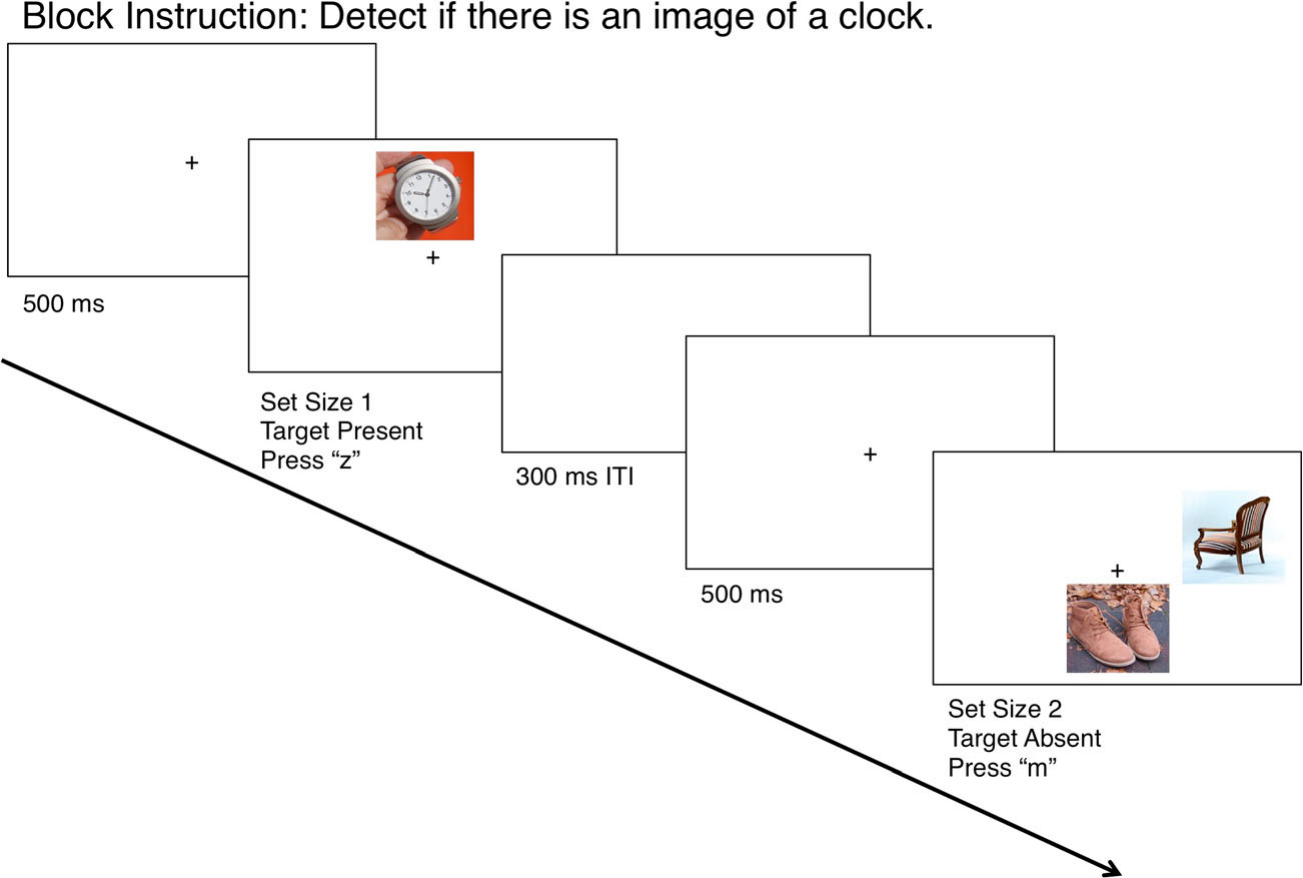
Sequence of events for two of the possible response displays in Experiment 1A, with unrelated target (i.e., clock and key) images. Additional response displays could present a set size of 1 or 2 with a target present or absent. Experiment 1B, with semantically related target images, followed the same sequence, but with images of locks and keys

The experimenter ensured that the observer fully understood the instructions before commencing the trials. On each trial, a fixation cross (Arial font size 12; 5 mm) appeared at the center of the screen for 500 ms, followed by the search display composed of one or two images. One-image trials presented the single image above, below, to the left, or to the right of the central fixation cross. Two-image trials presented all paired combinations of these locations. Each block contained equal numbers of one- and two-image search displays. Trials were further divided into *target-present* and *target-absent* trials, which were presented equally often in a pseudorandom order. Target-present one-image trials presented one image from the predefined target category/categories, whereas the target-absent one-image trials presented a single neutral category image. Target-present two-image trials presented one image from the predefined target category/categories and one neutral category image, and target-absent two-image trials presented two unique neutral images. Observers responded to the display by pressing “z” to indicate “target present,” or “m” to indicate “target absent,” as quickly and accurately as possible. A 300-ms intertrial interval passed, and the next trial began with the fixation cross in the center of the screen. Figure 1 schematizes the sequence of events in a typical trial.

Observers completed 288 trials organized into four blocks, two of which had two sets of 36 two-template trials presenting unpredictably either a key or a clock/lock target image on each target-present trial; the other two blocks were one-template blocks, containing two segregated sets of 36 trials in which all trials within a block contained only one type of target stimulus, with the type of target stimulus varying *predictably* across blocks. The intermixed, two-template blocks and segregated, one-template blocks were presented in a counterbalanced ABBA sequence, in which “A” denotes intermixed blocks for half of the observers and segregated blocks for the other half. Additionally, within the segregated blocks, the order of the target stimuli was counterbalanced across observers.

### Results and discussion

**Fig. 2:**
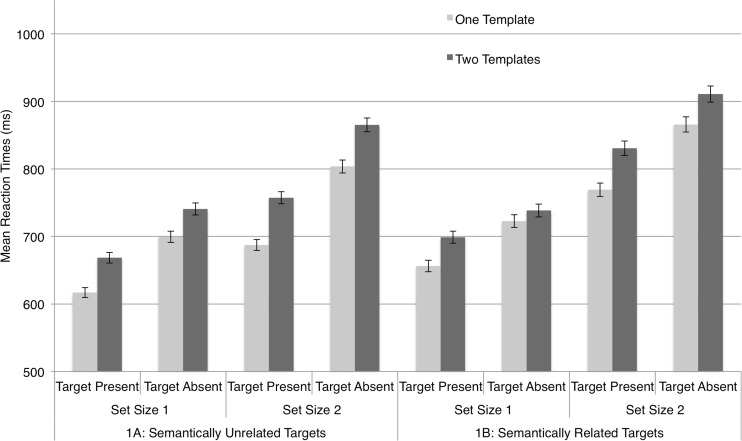
Mean reaction times for Experiments 1A (semantically unrelated target categories) and 1B (semantically related targets categories). The “two-template cost,” for each set size (one and two items) and for target-present versus target-absent trials, is indicated by the difference between the dark-shaded (two-template trials) and light-shaded (one-template trials) bars. Error bars = 1 *SEM*_paired diffs_

Accuracy was high (*M* = 97.19%, *SD* = 2.04%) and a stem-and-leaf diagram identified one outlier in Experiment 1B; this observer’s mean RT was more than three standard deviations from the group mean, so we excluded the observer’s data from the analysis. Figure 2 plots mean RTs across observers separately for Experiments 1A (unrelated targets) and 1B (semantically related targets). The results are grouped by one-template and two-template trial RTs for each set size and level of target presence. Visual inspection of the plot suggested that RTs were substantially slower for set size 2 than for set size 1, indicating that the target items did not effortlessly “pop out” at set size 2. The two-template costs in Experiment 1A versus 1B appeared to be roughly of the same magnitude.

To simplify our analysis, we first calculated the two-template cost, indicated in Fig. 2 by the difference in heights of neighboring one-template and two-template bars, for each observer for each condition (separately for set sizes 1 and 2 and target present and absent in Exps. 1A and 1B). Using two-template costs as the dependent variable, we ran a mixed, three-way analysis of variance (ANOVA), with one between-observers factor, Experiment (1A: semantically unrelated target categories, 1B: semantically related target categories), and the within-observers factors Set Size (response display of one vs. two images) and Target Presence (target present, target absent). This yielded no main effect of experiment [*F*(1, 37) = .681, *p* = .415], a marginal main effect of set size [*F*(1, 37) = 3.396, *p* = .073], and no effect of target presence [*F*(1, 37) = 2.161, *p* = .150]. Crucially, we observed *no* significant interactions (all *F*s < .344)—and, key for our purposes, no interactions involving the term experiment. Only the intercept term in this analysis [*F*(1, 37) = 29.225, *p* < .001], indicating a robust main effect of two-template cost across the conditions, was evident: The two-template conditions consistently yielded slower RTs than did the one-template conditions. However, these costs were significant in magnitude across all conditions for both experiments, as indicated by the absence of any interactions.

This pattern of results strongly contrasted with our previous findings with templates for rejection, in which two-template costs, which largely influence *attention guidance*, not recognition, were abolished by semantic relatedness. They tended to confirm our suspicion, first prompted by an experiment reported in a previous article (Daffron & Davis, 2015), that semantic overlap does not influence two-template costs for target templates.

Although the results of Experiment 1 revealed no such evidence of semantic guidance, we wondered whether such effects might yet be observed if we were to bolster the associations between the two semantically related target categories—locks and keys—using a (nonfinancial) reward-based training phase. By maximizing the degree of relatedness in observers’ perception, this manipulation might then permit them to specify overlapping semantic features of the categories and to minimize two-template costs. We had already adopted this strategy successfully to reveal the characteristics of templates for rejection in a previous project (Daffron & Davis, 2015, Exps. 2A and 2B), and were confident that, if such effects can be produced for target templates, this paradigm should reveal them.

## Experiments 2A and 2B

In Experiments 2A and 2B, we replicated the conditions of Experiment 1B, but now implemented one of two training phases prior to running those trials. For Experiment 2A (“training apart”), training rewarded observers when they assigned keys and locks to two different categories—presumably emphasizing their semantic differences. The training phase in Experiment 2B (“training together”), instead, rewarded observers when they assigned keys and locks to the same category—presumably emphasizing those categories’ shared semantic features. Negative feedback signaled an incorrect categorization in both training phases. If we were to find that two-template costs decreased in Experiment 2B, this would provide evidence that the semantic relationships between categories can affect search; we could then address whether those semantic features affected the guidance of attention—relative biasing of attention toward the target in two-item displays—or had solely affected the speed of target recognition. If, in contrast, the two-template costs were indistinguishable for Experiments 2A and 2B, it would be unclear whether this reflected an absence of target template semantic influences on performance or our manipulation being ineffective. One attractive feature of the task was that the stimuli for the two experiments were identical, so any difference in the two-template costs could not reflect bottom-up effects.

### Method

#### Observers

Forty paid observers (Exp. 2A, seven male, 13 female, 18–44 years of age; Exp. 2B, six male, 14 female, 20–30 years of age) from the University of Cambridge and the local area participated, having given informed, written consent. We expected that the effects of interest should be of similar size here to those in Experiment 1 and, hence, recruited the same sample size as for Experiment 2.

#### Materials

All of the computer equipment used in Experiment 2 was identical to that in Experiment 1. Experiment 2 consisted of a training phase and a testing phase, each with a unique set of stimuli. For the training phase, 152 images of objects, half of which depicted images that could be inserted into a corresponding counterpart (38 keys, 38 miscellaneous other “male” items), and the other half presenting the counterparts to the former images, which contained the niches for insertion (38 locks, 38 miscellaneous “female” objects). These images were presented individually in 150 × 150 pixel boxes in a randomized order at the center of the display. Observers categorized these images into “Category A” or “Category B” by pressing “c” or “n,” respectively, for each category on an attached Apple keyboard. Observer responses were followed by 1-s audio clips of crowd applause, for a correct response, or crowd booing, for an incorrect response, at a comfortable volume on the external computer speakers. In the testing phase, the observers in Experiments 2A and 2B performed the same task (with exactly the same stimuli) as described for Experiment 1B (see Fig. 1). The images used in each phase of the experiment were unique to that phase.

#### Procedure

*2A: Training locks and keys apart* This training phase of the experiment manipulated the strength of the semantic relationship between locks and keys to further separate them, by calling attention to their differing semantic features. Observers viewed centrally presented stimuli that were to be assigned to either of two categories. Observers were not instructed with information as to the correct categorization of these stimuli, only that they would learn through trial and error how to successfully complete the task. For Experiment 2A, the stimuli were assigned to two categories that would determine the observer’s correct response: “female” and “male.” The female category consisted of images containing niches for corresponding parts, including locks, as well as wall sockets, seat belt ports, computer USB insertion points, and other such objects with niches for insertion. The male-category objects were the corresponding counterparts to the niched images in the female category, which included keys, electrical plugs, seatbelt inserts, and so forth. Locks and keys therefore were assigned to differing categories, so as to *reduce* the perceived semantic relatedness of the two categories, at least within the context of our experiment.

*2B: Training locks and keys together* This training phase of the experiment manipulated the semantic relationship between locks and keys to *increase* the perceived semantic relatedness of the two categories. We expected that observers’ attention should now become focused on the shared semantic features of locks and keys. All of the stimuli in Experiment 2B were identical to those used in 2A, but correct categorization of the images now followed a different set of rules. Successful categorization in this experiment placed locks and keys together into Category A, and all other male and female objects into Category B.

Both training phases began with the same set of instructions, detailing to the observer their task of categorizing the centrally presented image into one of two undefined categories (Category A/Category B). Observers were instructed to learn through trial and error the correct categorization, with positive or negative visual and auditory feedback after each of their selections. After the attending experimenter was confident the observer could successfully complete the task, the experiment was initiated.

**Fig. 3:**
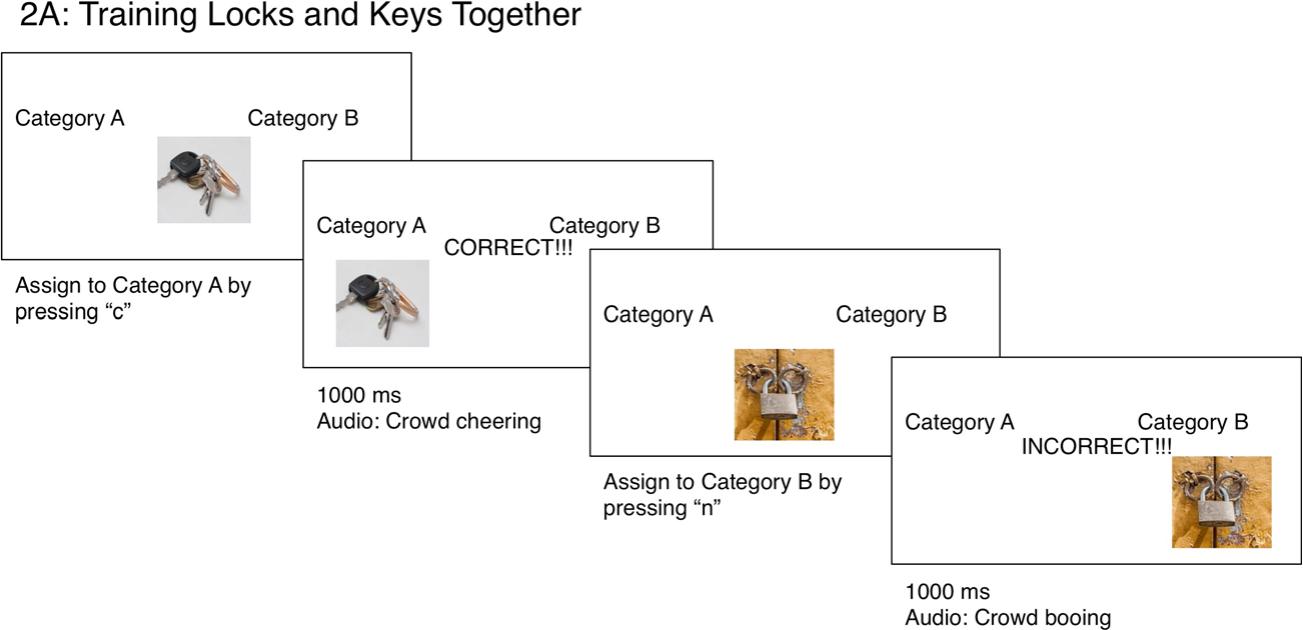
Sequence of events for the training phase of Experiment 2. This schematic shows the specific training for Experiment 2B, in which correct categorization places images of both locks and keys into “Category A,” with all other images in “Category B.” The sequence of events for Experiment 2A followed the same sequence of events, with “correct” feedback for categorizing locks and keys into separate categories with, respectively, other female and male items

Each trial was presented in the same format, with the text “Category A” centered 43 mm above and 90 mm to the left of fixation, and “Category B” at the corresponding position to the right of fixation, both in size 24-point Arial font; this text remained on the screen for the entirety of the training phase. Observers were instructed to classify the centrally presented image into either Category A or Category B by pressing “c” for Category A and “n” for Category B (i.e., the keys pertained to the boxes on the left and right of the screen, respectively). Observers received feedback to their response in the form of the word “CORRECT!!!” or “INCORRECT!!!” presented in the center of the screen in size 48 font with a corresponding positive (a crowd cheering) or negative (crowd booing) sound, for 1 s each. Figure 3 illustrates a typical trial’s sequence of displays in the training phase.

For Experiment 2A, positive feedback was given for categorizing “female” objects into Category A and “male” objects into Category B. All other responses received negative feedback. For Experiment 2B, positive feedback was given for assigning images comprising locks and keys into Category A, and all other stimuli into Category B, with other responses receiving negative feedback. Images remained on the screen until a response was made and feedback given. Upon completion of the training phase, observers completed a short questionnaire asking: “What images were categorized into Category A” and “What connection do you make between these images that would categorize them together?” The same questions were also asked of Category B.

After completing the training phase of Experiment 2A or 2B, all observers completed Phase 2, the search task testing phase. The task for Phase 2 replicated the conditions of Experiment 1B.

### Results and discussion

**Fig. 4:**
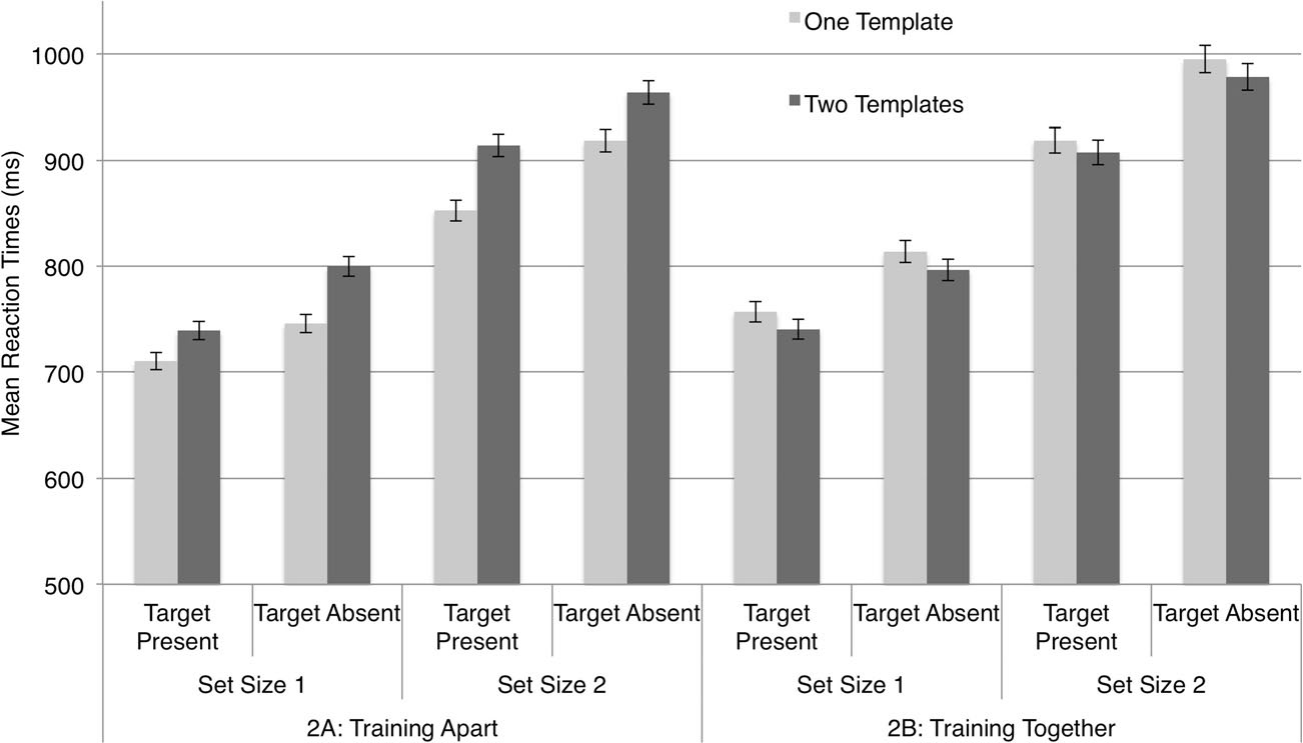
Mean reaction times (RTs) for Experiments 2A, training locks and keys apart (enforcing semantic distance), and 2B, training locks and keys together (increasing semantic overlap). RTs are presented separately for target-present and target-absent trials at each set size. Error bars = 1 *SEM*_paired diffs_

Accuracy was high (*M* = 95.68%, *SD* = 2.43%). A stem-and-leaf diagram identified two outliers, one in Experiment 2A and one in 2B, each with a mean RT more than three standard deviations from the experiment’s RT mean. Figure 4 plots the main RTs across observers separately for Experiments 2A (training apart) and 2B (training together) in one-template and two-template trials for each level of set size and target presence.

Visual inspection of the plot suggested that, as we had predicted, manipulating the strength of the semantic association before the testing phase of the experiment impacted the observers’ use of a semantic search template. Two-template costs were calculated separately for each observer and for all conditions, as in Experiment 1. These costs were analyzed in a mixed, two-way ANOVA, with the between-observers factor Experiment (2A, 2B) and the within-observers factors Set Size (response display comprising one or two images) and Target Presence (target present, target absent). This yielded a main effect of experiment [*F*(1, 36) = 5.612, *p* = .023]. We found no significant main effect of set size [*F*(1, 36) = 0.669, *p* = .419] or target presence [*F*(1, 36) = 0.001, *p* = .969]. Additionally, none of the interactions were significant (all *F*s < 1.382, n.s.).

The significant effect of experiment was investigated further by running two repeated measures ANOVAs on the two-template costs for Experiments 2A and 2B separately. This generated two separate intercept terms, one for each analysis, indicative of the two-template costs in each experiment. This term was significant for Experiment 2A [*F*(1, 18) = 5.174, *p* = .035], but not for Experiment 2B [*F*(1, 18) = 0.885, *p* = .359]. That is, the two-template cost evident in Experiment 2A (and in Exp. 1B, with semantically related stimuli) was not evident in Experiment 2B. As we expected, neither of these ANOVAs revealed other main effects or interactions (all *F*s < 1.872, n.s.). The overall two-template costs in Experiment 2A were of similar magnitude for set sizes 1 and 2 and target-present versus -absent trials; no such effect was observed in Experiment 2B. We concluded that, given sufficient task-specific training, the search templates in our task *could* specify semantic properties of the target categories to speed “recognition” of search items, but this would not influence the *guidance* of attention. We speculated (and hope to address this in future research) that this effect may have reflected the assignment of two object categories to a single category/response during training, speeding encoding of the target items as targets, rather than recognizing them as specific objects, in the subsequent test.

These findings contrasted with those for templates for rejection using a target-locating task (Daffron & Davis, 2015); in three out of three of those experiments, semantic relatedness abolished evidence of two-template costs that otherwise arose (exclusively) at set size 2. To confirm those findings in a search task demanding a binary target presence decision, as we employed here, we ran two further experiments replicating the conditions of Experiments 1A and 1B, but with different instructions. Observers now were instructed to *ignore* specified categories of objects, presumably employing templates for rejection to do so, rather than searching for them. The targets were undefined, other than that they were not either of the known types of nontargets.

The results from our previously published study on templates for rejection, using a target location task with only target-present trials, allowed us to make clear predictions for this new experiment’s target-present trials. Previously, two-template costs were only found for semantically unrelated categories at set size 2, whereas semantically related categories exhibited no cost at set size 2. Regardless of semantic relatedness, no costs were found at set size 1. This pattern suggested to us that the semantic relatedness of the two templates had influenced target guidance by the templates for rejection at set size 2; such effects on guidance would have been minimal at set size 1, in which the sole search item effectively cued attention to itself. We expected that such guidance effects could not operate effectively in this new experiment at set size 2 for target-absent trials, in which two nontargets were presented and relative guidance away from one item toward another could not greatly benefit performance. Accordingly, we could not make the same, clear predictions for target-absent trials in the new experiment.

## Experiments 3A and 3B

Experiments 3A and 3B were identical to Experiments 1A and 1B, with the important exception of reversed instructions: In Experiments 3A and 3B, observers were instructed to ignore specified nontarget object categories, as opposed to finding specified object categories. These experiments would thus explore the two-template costs associated with templates for rejection in the same target detection task, only this time the target would be unspecified, whereas the nontargets would be specified.

### Method

#### Observers

The power considerations were as for Experiment 1. Forty paid observers (in Exp. 3A, five male, 15 female, 18–32 years of age; in Exp. 3B, one male, 19 female, 19–28 years of age) from the University of Cambridge and the local area participated, after having given informed written consent. The observers for Experiment 3A were the first 20 (of 30) included as part of a supplementary analysis reported in Daffron and Davis (2015); these were matched with 20 new observers for Experiment 3B.

#### Materials

All of the computer equipment used in Experiment 3 was identical to that in Experiment 1.

#### Procedure

As with Experiment 1, Experiment 3 began with an instruction screen detailing the broad structure of the experiment. Observers learned that they would see three categories of images: keys, clocks (Exp. 3A)/locks (Exp. 3B), and “neutral” images. The task was to detect whether or not a neutral target item was present on each trial, and to ignore known nontarget categories of objects, either keys and clocks (Exp. 3A) or keys and locks (Exp. 3B). An additional instruction screen appeared before the initiation of each block to indicate the specific nontarget(s) that observers were tasked with ignoring in the following block. Thus, the procedure for Experiments 3A and 3B was identical to that in Experiments 1A and 1B, with only the exception of the word “ignore” replacing all instances in which the word “find” had been used in the previous experiments.

### Results and discussion

**Fig. 5:**
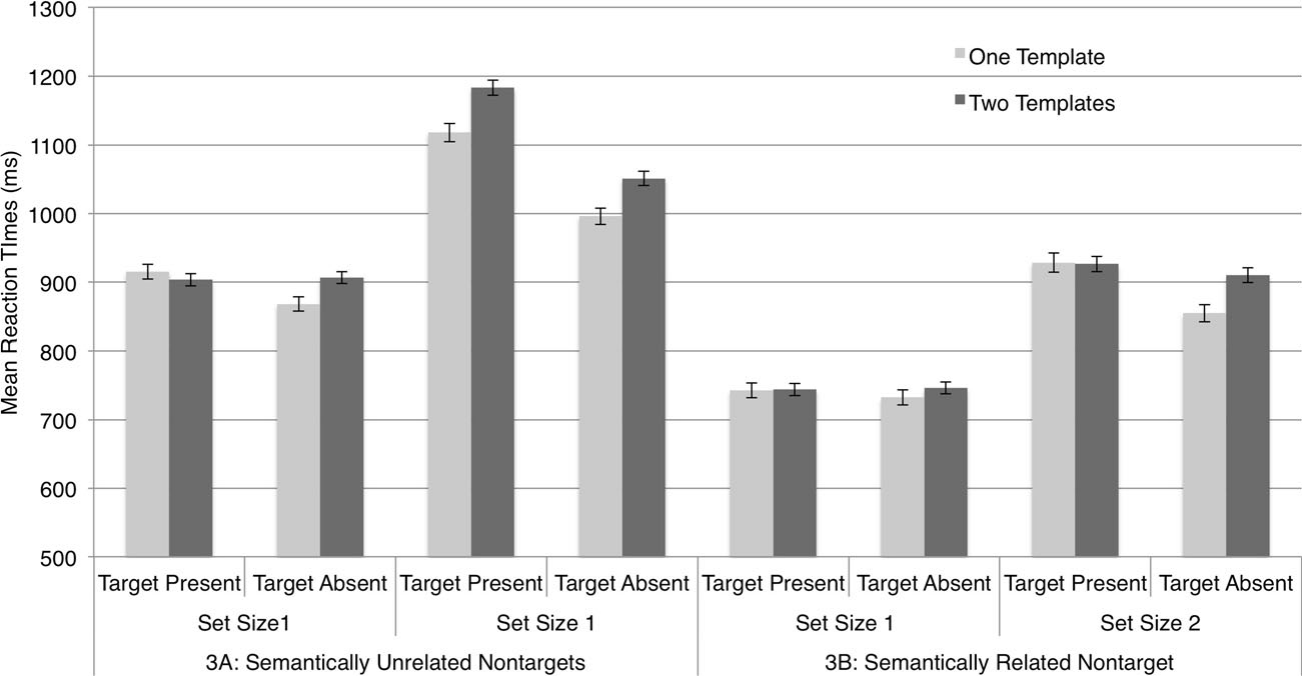
Mean reaction times (RTs) for Experiments 3A, semantically unrelated nontargets, and 3B, semantically related nontargets. RTs are presented separately for target-present and target-absent trials at each set size. Error bars = 1 *SEM*_paired diffs_

Accuracy was high (*M* = 93.56%, *SD* = 2.40%). A stem-and-leaf diagram identified no outliers. Figure 5 plots the main RTs across observers separately for Experiments 3A (semantically unrelated nontarget categories) and 3B (semantically related nontarget categories). Although the two-template costs in Experiments 1A and 1B were of equivalent magnitude, visual inspection of the mean RTs plotted in Fig. 5 indicates different relative patterns of results in Experiments 3A and 3B. Two-template costs were calculated for each observer for each combination of set size and target presence. These costs were then used as the dependent variable in a mixed, two-way ANOVA with the between-observers factor Experiment (3A, 3B) and two within-observers factors, Set Size (response display of one or two images) and Target Presence (target present or absent).

The ANOVA yielded no main effect of experiment [*F*(1, 38) = 0.688, *p* = .412], but main effects of set size [*F*(1, 38) = 7.591, *p* = .009] and target presence [*F*(1, 38) = 6.325, *p* < .016]. There were no two-way interactions (both *F*s < 1.333, n.s.), but the three-way interaction between set size, target presence, and experiment was significant [*F*(1, 38) = 5.636, *p* = .023]. To explore this interaction, the two-template costs were analyzed separately for target-present and target-absent trials in separate mixed ANOVAs. The conditions were split according to target presence, because, as we articulated in the discussion of Experiment 2, our conclusions from previous work made clear predictions for these.

**Fig. 6:**
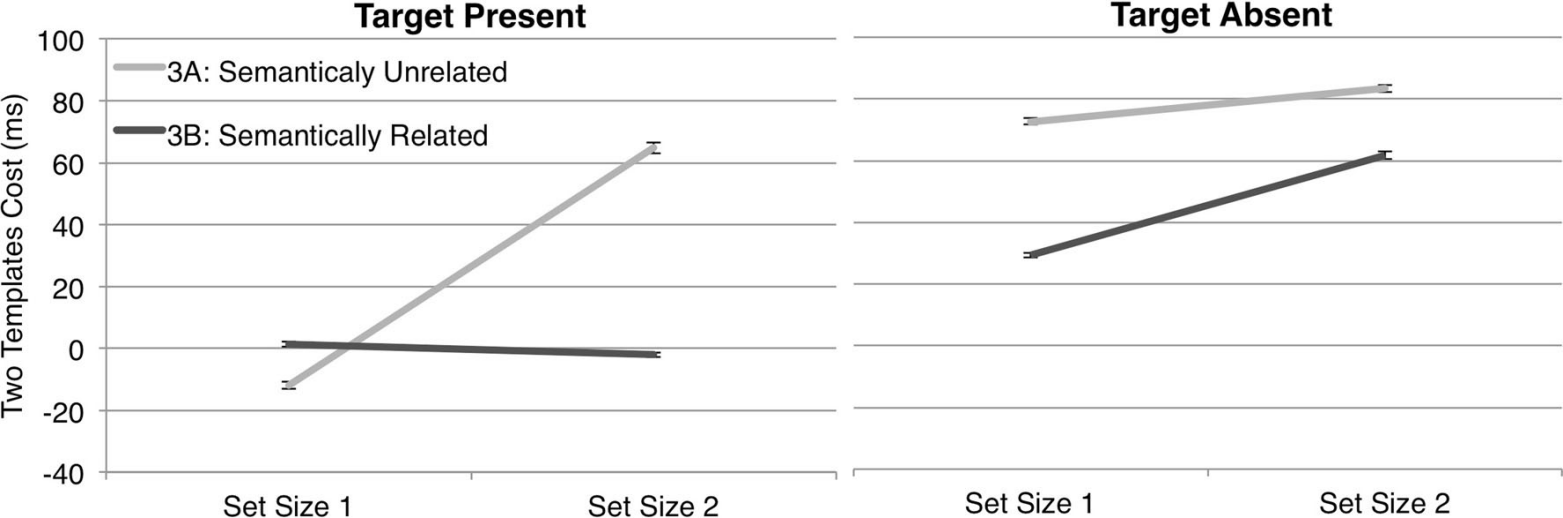
Two-template costs for Experiments 3A and 3B, displayed for set sizes 1 and 2 independently for target-present and target-absent trials. Error bars = 1 *SEM*_paired diffs_

To help visualize the effects in Experiment 3, Fig. 6 plots the two-template costs from Experiments 3A and 3B for target-present trials (left-hand plot) and target-absent trials (right-hand plot). Visual inspection of the left plot suggested that, as our previous work had led us to expect (Daffron & Davis, 2015), two-template costs in *target-present* trials were negligible at set size 1, and arose at set size 2 exclusively for unrelated targets (Exp. 3A), not for related targets (Exp. 3B). An ANOVA on target-present trial RTs confirmed these impressions, revealing no significant main effect of experiment [*F*(1, 38) = 0.991, *p* = .326], a main effect of set size [*F*(1, 38) = 5.400, *p* = .026], and crucially, a significant interaction between these two factors [*F*(1, 38) = 6.481, *p* = .015]. Two-template costs were lower at set size 2 for semantically related than for semantically unrelated categories [Exps. 3B (*M* = –2, *SD* = 67) and 3A (*M* = 65, *SD* = 139), respectively; *t*(19) = –2.067, *p* = .053], but this difference was reduced or absent at set size 1 (3B: *M* = 1, *SD* = 76; 3A: *M* = –12, *SD* = 97) [*t*(19) = 0.481, *p* = .636]. This was consistent with our previous conclusion that the semantic relatedness of two object categories can enhance attention guidance (away from nontargets) by templates for rejection in two-template trials. Also broadly consistent with this view, a corresponding ANOVA performed on target-absent trial RTs (Fig. 6, right-hand plot) revealed no main effect of set size [*F*(1, 38) = 2.975, *p* = .093] or experiment [*F*(1, 38) = 0.239, *p* = .628], and no interaction of these factors [*F*(1, 38) = 0.567, *p* = .456]. As we had expected, attentional guidance effects of the semantic templates for rejection did not greatly influence performance in target-absent trials.

We next assessed whether the pattern of results for templates for rejection in Experiments 3A and 3B differed from that from Experiments 1A and 1B for target templates, focusing particularly on target-present trials, which were physically identical in Experiment 1 versus 3. An ANOVA with two between-observers factors, Search Template Type (target template in Exp. 1, template for rejection in Exp. 3) and Semantic Relatedness of Categories (unrelated in Exps. 1A and 3A, related in Exps. 1B and 3B), and the within-observers factor Set Size (one vs. two items), revealed main effects of search template type [*F*(1, 75) = 7.671, *p* = .007] and set size [*F*(1, 75) = 6.827, *p* = .011], a marginal interaction between set size and semantic relatedness of categories [*F*(1, 75) = 3.556, *p* = .063], and importantly, a marginal interaction between all three factors [*F*(1, 75) = 3.639, *p* = .060]. This interaction between search template type, semantic relatedness of categories, and set size provided suggestive support for our view that target templates and templates for rejection are affected differentially by semantic relatedness, particularly at set size 2. However, even with 78 observers, our power to see this effect would likely have been limited.

To increase our confidence that such an effect was reliable, we next combined the data from Experiments 1 and 3 with RTs from corresponding conditions using a target location task (Daffron & Davis, 2015, with displays identical to those in Exps. 1 and 3 here) into one ANOVA. Set size 1 was removed, as it could clearly not drive the interaction term and was not featured in the target-locating task. Accordingly, in the resulting ANOVA, the factor Task (target location vs. target presence decision) replaced that of set size, with a total of 158 observers. This yielded significant main effects of semantic relatedness of categories [*F*(1, 157) = 6.613, *p* = .011] and task [*F*(1, 157) = 6.642, *p* = .011]. Importantly for our conclusions, there was also a significant interaction between the semantic relatedness of categories and the search template type [*F*(1, 57) = 4.305, *p* = .040], but no higher-order interaction that might threaten our interpretation of it. Semantic relatedness reduced two-template costs significantly more for templates for rejection than for target templates.

We concluded that the semantic relatedness of specified search categories enhances attention guidance by templates for rejection, but found no evidence of such effects for target templates. Target templates, in these analyses, do not appear to guide attention on the basis of semantic properties; if such were the case, they must presumably do so on the basis, primarily or exclusively, of visual information. To assess this, in a final experiment, we next turned our attention to whether guidance of search *in our task* could be influenced when target templates specified visual rather than semantic information. In this new experiment we adopted the same logic and approach as in Experiments 1 and 2, but now examined two-template costs in relation to visual rather than semantic properties.

In this new study, the target stimuli were always photographs of keys—this category was chosen because keys do not have a clear canonical orientation (unlike, e.g., clocks or locks), and because we had used them in the previous three experiments. Hence, the broad semantic category of the target items remained constant across conditions. What altered across conditions was how much visual information about the target was specified by a cue appearing at the beginning of each trial. We created three conditions: In the first condition, “one-template,” the cue was of two identical target images, presented *at the same angle of rotation* at which the target would be presented. Observers therefore needed only establish a single search template for one specific set of visual features (colors, spatial frequency spectra, etc.) at one specific orientation. In a second condition, “two-angles,” the cue again presented two images of the target that would appear now *at two different angles*; the target was presented at either of those angles of rotation. If the particular orientation of the target object was essential to their search template for the recognition of items or in the guidance of attention, then performance should improve in the one-template condition relative to the two-angles condition. In a third condition, “two-exemplars,” the cue consisted of two different images of keys, each presented at the same angle of rotation as the target that would subsequently appear. Accordingly, if the ability of observers to specify the visual features (colors, spatial frequency spectra, etc.) with their search template, irrespective an image’s particular orientation, was important to recognition or guidance processes, we should observe improved performance in the one-template relative to the two-exemplars condition. Inclusion of both the two-angles and two-exemplars conditions within the same experiment also permitted us to consider direct comparisons of those two conditions, which as we discuss below, proved instructive.

## Experiment 4

In Experiment 4, the targets were no longer specified in terms of their broad semantic categories, but rather in terms of specific images. In “one-template” trials, observers were presented, in the “targets” (i.e., cue) display on each trial, with two identical examples of the particular target image they would be asked to search for in the subsequent search display. There were two types of two-template trials: In both cases, two different possible targets were presented to the observer in the “targets” display, either of which might be the target in the subsequent search display. In the “two-angles” condition, the two items presented in the “targets” display were the same image of keys, but presented at two different angles of rotation. In the “two-exemplars” condition, the two items presented in the “targets” display were two different images of keys.

### Method

#### Observers

Because we had no indication of the size of the effect we might expect, and no support from our previous studies regarding the pattern of results to expect, we estimated sample size on the basis of Cohen’s *f* = 0.25 for six measurements to yield 90% power. Twenty-four observers (11 male, 13 female, 19–41 years of age) from the University of Cambridge and the local area gave written, informed consent and were paid for participating.

#### Materials

All experimental materials were identical to those used in Experiments 1, 2, and 3, with the exception of the stimuli: The stimuli used in Experiment 4 were cropped to form circular images with a 150-pixel diameter. These circular images appeared in 150 × 150 pixel boxes with a black fill, to maximize the similarity of these images to the search items in Experiments 1, 2, and 3. Circular images were chosen for this experiment because the paradigm required rotation of the images. The images were surrounded by the filled-in boxes to keep continuity of the stimuli among experiments, as well as to keep the edges of the images standardized. The observers viewed 384 randomly selected neutral images, 265 key images rotated 0° (their original orientation), 32 key images rotated 45° (16 clockwise, 16 counterclockwise), and 32 key images rotated 135° (16 clockwise, 16 counterclockwise). Experiment 4 consisted of three types of blocks of trials, pertaining to the one-template, two-angles, and two-exemplars conditions, each of which was run four times. Images were repeated only once, in the second half of the experiment.

#### Procedure

The experiment began with an instruction screen detailing the overall structure of the experiment. Observers learned they would be presented with a “targets” display presenting two target images in the center of the screen. They were instructed to look at the images and to search for those images in a subsequent search display. The observers’ task was to detect whether one of the target images was presented in the response display as quickly and accurately as possible. For the one-template condition, the two targets were identical key images, both rotated 0°. The two central target images in the two-angles condition were identical images presented at two different angles; one image was rotated 0°, and the other image was rotated either 45° or 135° from center (equal representations of both rotations). The two-exemplars condition’s central target images were two unique images presented rotated 0°. Additional instructions were provided at the initiation of each block, to communicate to the observer which type of block was about to commence.

**Fig. 7:**
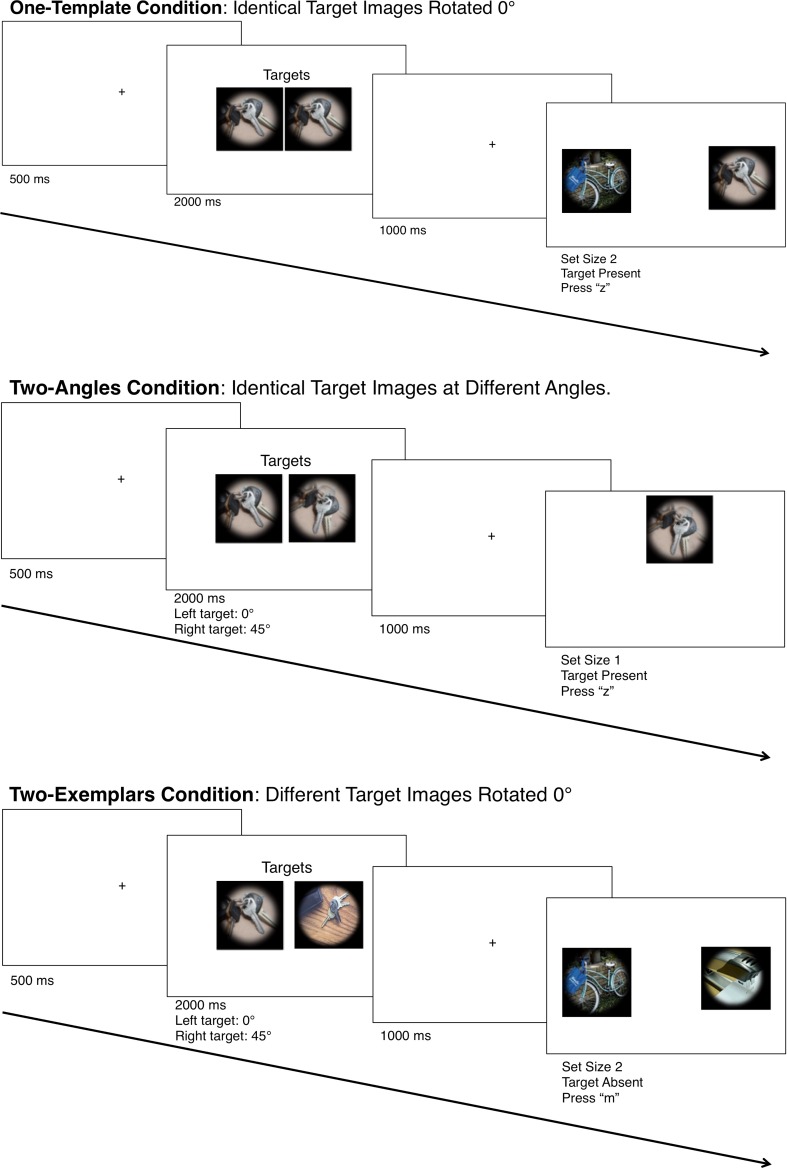
Sequences of trials in Experiment 4 for the different conditions. None of the images were repeated across the conditions; the images above are only demonstrated in schematics to directly compare the differences among the conditions

The experimenter ensured that the observer fully understood the instructions before commencing the trials. Figure 7 schematizes the sequence of events in typical trials. On each, a fixation cross (Arial font size 12; 5 mm) appeared at the center of the screen for 500 ms, followed by two centrally presented images with the word “Targets” appearing above them (Arial font size 24; 8.2 × 1.8 mm), centered at 962 × 420 pixels (each box beginning 22.5 mm from left and 10.5 mm from top of the image). The “Targets” text and target images remained on the screen for 2,000 ms, followed by a blank screen for 1,000 ms, and finally the search display, which remained on the screen until a response was made. As in Experiments 1, 2, and 3, the search display comprised one or two images, with a target presented on half of the trials. See the Method section of Experiment 1 for further details. Observers responded to the display by pressing “z” to indicate “target present” or “m” to indicate “target absent,” as quickly and accurately as possible. A 300-ms intertrial interval passed, and the next trial began with the fixation cross in the center of the screen.

Observers completed 384 trials organized into 12 blocks, with each block type presented four times. Blocks for the one-template condition presented two known targets at a common known angle, thus requiring only one template. Blocks for the two-angles condition presented two known targets at differing angles; the target’s precise angle of presentation was unpredictable. If recognition or guidance processes in the search task were sensitive to observers being able to specify the target’s angle of rotation with their search template, then this condition would require the observer to establish two search templates concurrently, and hence should incur performance costs. Blocks for the two-exemplars condition presented two different images, thus also requiring two templates, as either image could be presented as the target. The run order was determined by a Latin square configuration, with equal numbers of observers running the repeated ABC, BCA, and CAB orders.

### Results and discussion

**Fig. 8:**
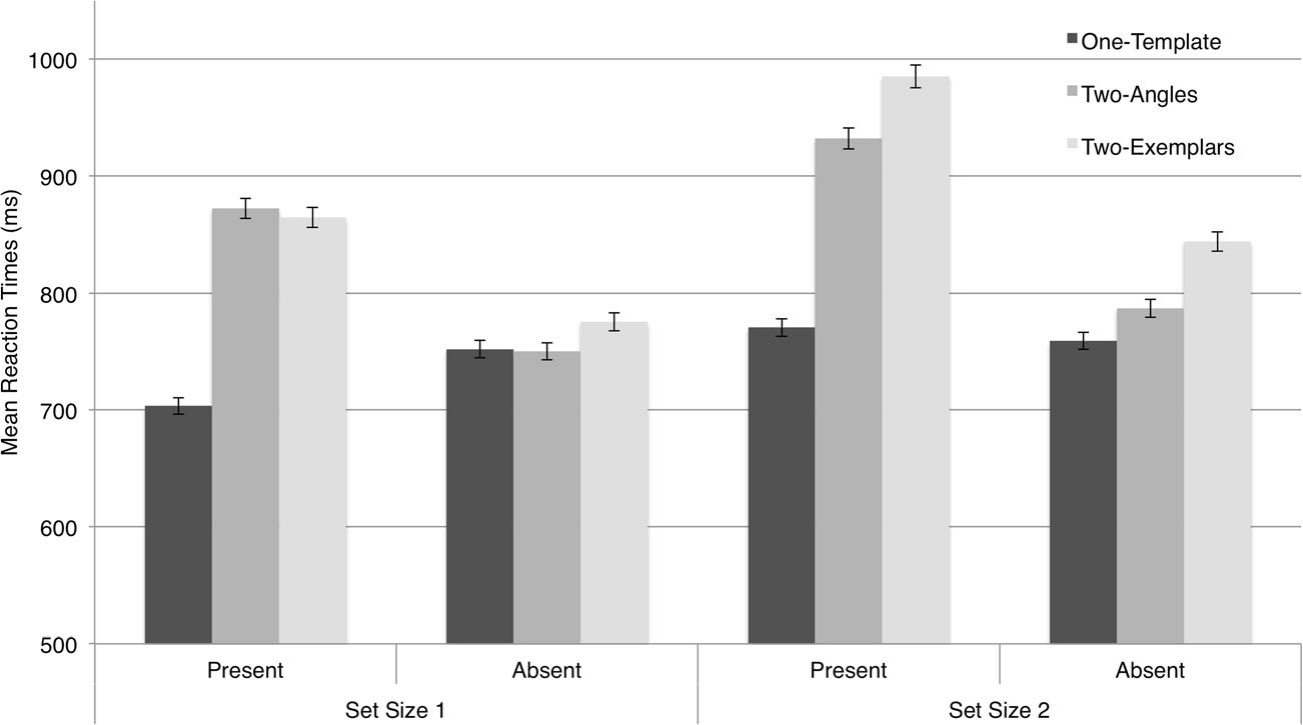
Mean reaction times for the three conditions in Experiment 4. RTs are presented separately for target-present and target-absent trials at each set size. Error bars = 1 *SEM*_paired diffs_

Note that the dependent variable was RT rather than two-template costs, since there was only one one-template condition to be related to both the two-angles and two-exemplars conditions. Accuracy was high (*M* = 96.54%, *SD* = 2.14%). A stem-and-leaf diagram identified one outlier; this observer was excluded for having less than our criterion of 80% accuracy (67.97% correct). Figure 8 plots the main RTs across observers separately for the one-template, two-angles, and two-exemplars conditions, for set sizes 1 and 2, and for target-present and target-absent trials. A within-observers repeated measures ANOVA was conducted on the mean RTs from Experiment 4 for the within-observers factors Set Size, Target Presence, and Condition (one-template, two-angles, two-exemplars). Significant main effects were apparent for all within-observers factors: Set Size [*F*(1, 22) = 28.375, *p* < .001], Target Presence [*F*(1, 22) = 13.039, *p* = .002], and Condition [*F*(2, 21) = 22.544, *p* < .001]. There were several significant interactions between the factors, including interactions between set size and target presence [*F*(1, 22) = 9.301, *p* = .006], set size and condition [*F*(2, 21) = 9.546, *p* = .001], and target presence and condition [*F*(2, 21) = 38.965, *p* < .001]. We observed no three-way interaction [*F*(2, 21) = 0.467, *p* = .633].

**Fig. 9:**
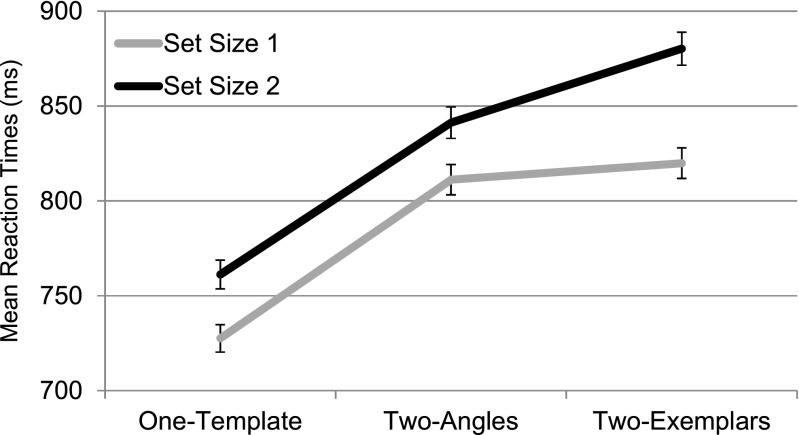
Reaction times for the three conditions separately for set sizes 1 and 2, irrespective of target presence, to highlight features in the interaction of interest. Error bars = 1 *SEM*_paired diffs_

We first considered the interaction of most interest—that between condition and set size, irrespective of target presence. Figure 9 plots the RTs of these conditions to clarify performance between the particular conditions of interest. We first noted that, *irrespective of set size*, both the two-angles condition (*M* = 835, *SD* = 225), and the two-exemplars condition (*M* = 865, *SD* = 242), yielded slower RTs than did the one-template condition (*M* = 742, *SD* = 203) [*t*(22) = –4.248, *p* < .001, and *t*(22) = –6.969, *p* < .001, respectively]; that is, both yielded a two-template cost relative to the one-template condition, whereas the two conditions yielded similar overall RTs [*t*(22) = –1.44, *p* = .163]. This similar relationship was particularly evident at set size 1, a point we discuss immediately below.

We concluded that when observers could not specify, with their target search template, either the exact angle of the target (as in the two-angles condition) or the target’s particular image features (colors, spatial frequency makeup, etc., as in the two-exemplars condition), this generally slowed recognition of the targets as targets (or of nontargets as nontargets). Thus, for recognition processes, we found a general effect of increased uncertainty about the target that was of similar magnitudes in the two-angles versus the two-exemplars condition, evident at set size 1. On the other hand, the effects of such uncertainty on the *guidance of attention* should be expressed in greater increases in RTs for set size 2 than for set size 1 in the two-angles or two-exemplars conditions, relative to the one-template condition. Note that no such differences seem to arise for the two-angles versus one-template condition, as is evident in the parallel slopes for those two conditions (reflecting the fact that the RT increments at set size 2 versus 1 were the same for the two conditions’ data points). Calculating the slowing in milliseconds at set size 2 versus 1 for each observer in one-template (*M* = 37, *SD* = 71) and two-angles (*M* = 48, *SD* = 59) conditions, we found no evidence that the effects differed in magnitude [*t*(22) = –0.789, *p* = .439]. Though the efficiency of recognition processes differed in the one-template versus the two-angles condition, the efficiency of guidance did not.

The two-exemplars and one-template conditions also seemed to differ, as we noted, in terms of recognition processes (more efficient for one-template). However, here we did find evidence that the effects of set size also differed between the two conditions. When we calculated the slowing in milliseconds at set size 2 versus 1 for each observer in the two-exemplars condition (*M* = 95, *SD* = 69), comparing these to the one-template condition (*M* = 37, *SD* = 70), the former effect was larger [*t*(22) = –4.170, *p* < .001], raising the possibility that the guidance of attention may have been less efficient in that condition. However, on the basis of the logic outlined in the introduction, such a conclusion cannot be drawn confidently, given that there were also differences between the conditions at set size 1, since only effects observed exclusively at set size 2 can be ascribed, with any confidence, to attention *guidance* rather than recognition. Fortunately, however, a final comparison, between the effects of set size in the two-angles versus two-exemplars conditions, can help reveal influences of search templates on attention guidance in this task.

At set size 1, the two-angles (*M* = 811, *SD* = 236) and two-exemplars (*M* = 820, *SD* = 241) conditions were slowed roughly equal, relative to the one-template condition—the RTs for the two conditions were not found to differ [*t*(22) = –0.416, *p* = .681], and indeed were very similar. Accordingly, we can assume that the influences on recognition processes were similar for the two manipulations in the two-angles versus the two-exemplars case. In contrast, at set size 2, the RTs for the two-angles (*M* = 860, *SD* = 216) and two-exemplars (*M* = 914, *SD* = 249) conditions *did* differ [*t*(22) = –2.367, *p* = .027], being slower for the latter. These different patterns in the two conditions reflected greater slowing of RTs, at set size 2 relative to set size 1, for the two-exemplars (*M* = 121, *SD* = 98) than for the two-angles (*M* = 60, *SD* = 85) condition [*t*(22) = –2.320, *p* = .030]. Accordingly, it appeared that comparison of the two conditions yielded differences only at set size 2, not set size 1, our discussed criterion for detecting differences in the guidance of attention that cannot be plausibly be attributed to differences in the speed of recognition. We concluded that whereas the manipulation of target uncertainty in two-angles versus the two-exemplars condition affected recognition processes equivalently, it differentially affected guidance processes. When observers were uncertain as to which of two exemplars (with different visual features) the target might be, the guidance of search was more markedly impoverished than when observers were uncertain as to the angle at which a given target might be presented.

Two other significant interactions were reported above. First, the Condition factor interacted with Target Presence. This reflected the fact that any differences between conditions that were not set size dependent (and that, largely, we attributed here to effects on recognition) were evident in target-present trials, rather than in target-absent trials. This was to be expected, given that the manipulation of target search templates was unlikely to affect the recognition of not-specified nontargets without target items in the target-absent trials. Second, the interaction between set size and target presence reflected the fact that for the two-angles and two-exemplars conditions, but not for the one-template condition, the RTs to target-present trials were slower than the RTs to target-absent trials, presumably reflecting particular uncertainty about the target items in this experiment.

Together, the results from Experiment 4 suggest that when an observer can specify, with a target template, the specific exemplar of a target (its colors, texture, etc.), this will guide search more efficiently toward the target than when a target may be either of two exemplars. However, specifying the target’s angle of presentation and its features, or semantic information about the target, will not (measurably, in our study) affect the guidance of attention; these features will only increase efficiency of the recognition of individual search items.

## General discussion

In four experiments, reported here, we examined patterns of “two-template costs,” ascribing these costs either to slowed “recognition” or to impoverished guidance of attention. In Experiments 1 and 2, when observers established target templates to search for broad categories of objects, the two-template costs were similar for two-image displays versus one-image displays, consistent with an influence of those target templates on recognition rather than the guidance of attention. The semantic relatedness of possible target categories did not influence the two-template costs in Experiment 1, in agreement with our previously reported findings (Daffron & Davis, 2015, Exp. 6). However, training to enhance the related categories’ apparent relatedness in Experiment 2 did abolish these costs—an effect that arose equally at the two set sizes, again consistent with effects on recognition processes, not on attention guidance. We speculated that this effect of training may have arisen due to assignment of the two categories to a single response. Finally, the results from Experiment 4 identified a type of two-template cost for target templates that appeared at set size 2, not set size 1, consistent with the guidance of attention. These costs suggested that target templates may specify a target’s visual features (irrespective of its viewing angle) to guide attention. Further specifying the target’s angle of presentation did not enhance this guidance.

Our conclusions are broadly consistent with models of search, such as Wolfe’s Guided Search model (e.g., Guided Search 4.0; Wolfe, 2007; Wolfe et al., 2011), in which loosely bound sets of visual features guide search, while objects’ more complex, perhaps semantic, features can contribute to the speed with which they are recognized. However, our findings contradict previous claims that target templates specify the semantic properties of objects to guide attention in natural scenes (e.g., Belke et al., 2008; Moores et al., 2003). Admittedly, as we discussed earlier, our sample sizes of 40 observers per experiment did not allow us much confidence of having detected effects of Cohen’s *f* = 0.25 or smaller (a power of only around .75, even given a very liberal alpha). However, the overall pattern of effects across the experiments also held for the same stimuli when we had used a target-locating task (Daffron & Davis, 2015); if any effects of semantics in target templates were operating, they were presumably small and unreliable. If this is indeed the case, previous evidence for claims that target templates specify semantic information may have reflected the multiple presentations of particular target items in those studies. Thus, the previous studies may have unwittingly tapped the target templates that specified those particular visual features, rather than semantic features, as was intended. In our work, no image was presented more than once (other than in Exp. 4).

As we have noted throughout, these conclusions for target templates differ markedly from those for templates for rejection. Daffron and Davis (2015) found that templates for rejection (i.e., specifying nontargets’ properties) yielded two-template costs only at set size 2, and only for semantically unrelated categories, a pattern consistent with templates for rejection specifying nontargets’ semantic features, and this solely enhancing attention guidance (not recognition processes). Do these differences between our results for target templates versus templates for rejection reflect fundamentally different processes in the two cases, or do the two types of template effects differ only in degree, in the sizes of the effects? We know from our ANOVAs that for set size 2 target-present trials (across Exp. 3 and Daffron & Davis, 2015, Exp. 1), costs were greatly reduced—and seemingly, abolished—for semantically related categories (mean, –1.8 ms) versus semantically unrelated categories (mean, 51.7 ms) or for the corresponding target template conditions (37 ms). However, null-hypothesis testing tools are ill-suited to provide evidence for the absence of a two-template cost for semantically related categories. Accordingly, using a Bayesian analysis (Dienes, 2008) of these semantically related trials (target present, set size 2), we compared the evidence for a mean effect of zero versus 25 ms (half the effect found for semantically unrelated trials). This analysis yielded a Bayes factor of 0.32, providing statistical evidence for the null hypothesis. That is, the effect for related categories is more likely to be 0 ms than even a small effect of 25 ms. It would appear that two-template costs for templates for rejection are effectively zero for semantically related categories.

This absence of two-template costs for semantically related but physically distinct stimuli, with templates for rejection, is perhaps more remarkable than we recognized when we first reported this (Daffron & Davis, 2015). The absence of any cost suggests that in the one-template trials of those experiments, when observers knew a single category’s visual features as well as its broad semantic features, they were completely unable to exploit their knowledge of those visual features to enhance their guidance of attention. This result may make more sense in the context of another recent finding (Becker, Hemsteger, & Peltier, 2016), that templates for rejection cannot specify simple physical features to guide attention away from nontargets, even for simple abstract displays (in contrast, target templates can specify such simple features very effectively). Perhaps, we speculate, templates for rejection may only be able to effectively guide attention by specifying the high-level properties of stimuli. In contrast, target templates clearly do specify targets’ visual features to guide attention—doing so very effectively—yet there is no evidence that they can specify semantic features for this purpose.

As well as the guidance of attention, templates may independently speed the recognition of search items (in parallel or in serial), or decisions as to their target/nontarget status. For target templates, there appears to be no strong effect of semantic relatedness on this effect, but a fairly substantial effect of grouping two categories into one response category during training. Unfortunately, we have not yet tested this training effect for templates for rejection; there are, as yet, too few constraints on the interpretation of this finding to support further speculation.

Overall, templates for rejection specify semantic properties to guide search, yet do so without influencing “recognition,” whereas target templates specify (largely or exclusively) visual features, only showing evidence of semantic influences, on recognition, following training. How can these entirely opposing findings be integrated within a single cognitive architecture? Certainly, the working assumption that suppressing attention to nontargets is like guiding attention to targets, but with the sign (+ or -) reversed, is not plausible. Here, we briefly consider two candidate accounts: “late-versus-early” and “acuity” views.

The differences between target templates and templates for rejection may reflect, as we speculated above, a tendency for templates for rejection to select on the basis of higher-level properties, but for target templates to select bundles of visual features to guide attention. This “late-versus-early” selection account is complicated by our finding that target templates showed reduced two-template costs following associative training, suggesting that not all effects of target templates are based on the visual features of the stimuli. However, alterations to “recognition” processes may yet prove to be changes to decision criteria rather than to perception. In such a case, the “late-versus-early” view might explain our findings well.

A second candidate view is motivated by reconceptualizing two-template costs in terms of the benefits of knowing specific information in one-template trials, rather than the costs of applying two templates in two-template trials. Suppose that templates for rejection can only select a broad swathe of semantic features covering the categories of both locks and keys (akin to a wide spotlight in spatial attention, but across semantic space). This might mean that they could not select the semantic features of either category in isolation, explaining why knowing the specific category of the target (either a lock or a key) in one-template trials would not benefit the observer (and hence, no two-template cost would be observed)—the template could not specify information with sufficient precision. In contrast, target templates may be able to specify the precise category of object (either locks or keys) in one-template trials, and hence benefit performance relative to two-template trials (yielding a two-template cost). This “acuity” account is intuitive, but it also suffers from a serious shortcoming: failing to explain why two-template costs for target templates depend very little on set size, whereas templates for rejection showed no costs at set size 1 in any of our previous studies.

To summarize our discussion here, templates for rejection seem to differ greatly from target templates—as much as is possible within the constraints of our task. This may be because templates for rejection either specify higher-level information (the “late-versus-early” account) or cannot specify information as precisely (the “acuity” account) as target templates. Although a complete understanding of these differences still eludes us, our findings provide strong evidence of marked differences between the two types of templates. Certainly, our findings further undermine “search-and-destroy” models, in which items are ignored by first searching for them, then suppressing attention to them. Those models assume that ignoring an object involves the same process of attention guidance toward the target as searching for an object—in essence, that templates for rejection do not exist. Our view is that search-and-destroy models are no longer tenable. Instead, top-down templates that guide attention toward known targets differ fundamentally from those that guide attention away from known nontargets.
